# Supplementary material for: The Splicing Factor OsSCL26 Regulates Phosphorus Homeostasis in Rice
Source: Plants (Basel). 2023 Jun 15;12(12):2326. doi: 10.3390/plants12122326 (PMC10302782; doi:10.3390/plants12122326)
Supplement: Supplementary file 1 [file plants-12-02326-s001.zip › plants-2416291-supplementary.pdf]

Table S1. Primers used in this study

Primer Name	Sequence (5′-3′)
**gRNA of *OsSCL26***	
SCL26 -gRNA-F	GGCAGATGATCTACGAAGACCATT
SCL26 -gRNA-R	AAACAATGGTCTTCGTAGATCATC
**Mutant Identification**	
scl26 -jianding-F	GAGCTTGGTTTGCAATGCCAAAA
scl26 -jianding-R	CTGCTGACCTTGTACTCATCAGAGT
***OsSCL26* expression pattern**	
SCL26 -RT-F	GCACTATTCAAGCAGGTCTC
SCL26 -RT-R	TGGGTCCTAAGCTCCTCAC
**subcellular localization**	
SCL26-PHB-GFP-F	ctctctctcaagcttggatccATGGGAAGAGGCTATGATTATG
SCL26-PHB-GFP-R	agaaccacgggtcatgagctcCCGGCTAACAGAAAGAGACC
SCL26-GFP-F	atttacgaacgatagccatggtcATGGGAAGAGGCTATGATTA
SCL26-GFP-R	gtaccgtcgactgcagaattcCCGGCTAACAGAAAGAGACCT
**Histochemical localization of GUS expression**	
SCL26-GUS-F	caggtcgactctagaggatccACCTGCTCATCCCGTACACCT
SCL26-GUS-R	cctcagatctaccatggtaccTTTTTCCTGTAAGCTTAGATCAC
**PSI qRT-PCR**	
SPX1-RT-F	CATGCTGGACCAGCTTCTACC
SPX1-RT-R	TATTCCACAGTTCCTGCTGCTCC
SPX2-RT-F	GGCAGGATGAACCGGGGAGGTG
SPX2-RT-R	CATTTTTACAGCAGGTGGGAAAC
SPX4-RT-F	AATCCCTTGTCGCTCGC
SPX4-RT-R	TGCAAAGACGGATCATTCAT
OsSPX-MFS1-F	GCGATTCTTGGGTGTACTGT
OsSPX-MFS1-R	CGTGAAAGCAACGACAGGTT
OsSPX-MFS2-F	CCTCCTGAATGTAACCCTG
OsSPX-MFS2-R	AGGAACTGTGTCAACTGCTT
OsNLA-L-F	AGGCGAAGTGTCCTGTATGC
OsNLA-L-R	TCCAGTAATCCTTGCTCCTTGT
OsPT1-F	CGCTTCCGTACGAGTGGTAGT
OsPT1-R	GGTTCTTTCAAATCCAGGGAAA
OsPT8-F	TCCAGAAGGACATCTTCACCAGCA
OsPT8-R	ATGTCGATGAGGAAGACGGTGAAC
OsPT14-F	TTTTTCAGGATTGGCCAGAGTAA
OsPT14-R	AACGGACGAACAAGCTGGAGT
OsPT22-F	TGGGTGTTGGTGTGGATATCTGCT
OsPT22-R	TAGCTTGCTGAAAGGAGGGACCTT
OsACTin-F	CAACACCCCTGCTATGTACG
OsACTin-R	CATCACCAGAGTCCAACACAA
**Validation of AS**	
OsNLA-L-AS-F	TTCGGTGCAATATATGAAGA
OsNLA-L-AS-R1	TAAAGAACATTTGATCACACA
OsNLA-L-AS-R2	CCTTAGTAAGTTATGTGCATG
SPX-MFS2-AS-F	GAACCGCAGGTACATCAGCGACTGT
SPX-MFS2-AS-R1	ACGTAATCCAAGCCAAGCAC
SPX-MFS2-AS-R2	CTCTTGCCGAGCCAGACCACAT
